# Fabrication of Bacteria Cell Arrays with Optical Vortex Laser-Induced Forward Transfer

**DOI:** 10.1021/acsomega.5c03716

**Published:** 2025-08-05

**Authors:** Srinivasa Rao Allam, Ken-ichi Yuyama, Kaito Sato, Mitsumasa Hanaoka, Takashige Omatsu

**Affiliations:** † Graduate School of Engineering, 12737 Chiba University, 1-33 Yayoi-cho, Inage-ku, Chiba 263-8522, Japan; ‡ Molecular Chirality Research Centre, Chiba University, 1-33 Yayoi-cho, Inage-ku, Chiba 263-8522, Japan; § Institute for Advanced Academic Research, Chiba University, 1-33 Yayoi-cho, Inage-ku, Chiba 263-8522, Japan; ∥ Department of Chemistry, 12936 Osaka Metropolitan University, 3-3-138 Sugimoto, Sumiyoshi-ku, Osaka 558-8585, Japan; ⊥ Graduate School of Horticulture, Chiba University, 1-33 Yayoi-cho, Inage-ku, Chiba 263-8522, Japan; # Department of Electrophysics, National Yang Ming Chiao Tung University, 1001, Daxue Rd. East Dist., Hsinchu 300093, Taiwan

## Abstract

The optical vortex laser-induced forward transfer (OV-LIFT) technique enables the direct print of well-aligned dots with high spatial resolution and high positional accuracy. In this work, we demonstrate the direct printing of a 2-dimensional biomaterial (cyanobacteria cells) dot array using the OV-LIFT technique. The number of bacteria and size of the printed dots were controlled by simply adjusting the thickness of the donor film and the numerical aperture (NA) of focusing optics. The cell viability (∼90%) of cyanobacteria cells in the as-printed dots with OV-LIFT is significantly higher than that (>63%) achieved when using a conventional laser-induced forward transfer (LIFT) process. This demonstration highlights the viable application of OV-LIFT to the fabrication of freeform two- and/or three-dimensional printed microchannels for advanced applications such as light-harvesting devices based on biomaterials.

## Graphical abstract

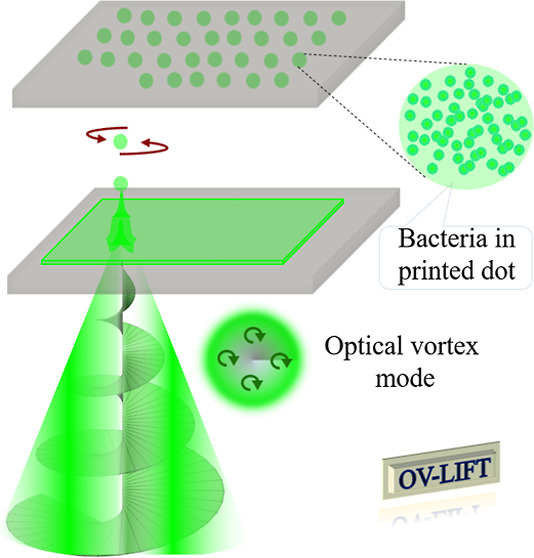

## 1 Introduction

Across the fields of printed electronics and photonics, there is a growing demand for a cost- and resource-saving, on-demand direct printing technique. For many years, nozzle-based inkjet technologies have met these needs, being capable of fabricating structures with exotic designs and features (in contrast to using conventional etching processes).^1–3^ However, inkjet technologies still have capacity limitations due to nozzle-clogging effects;^4,6^ for instance, inkjet technologies still struggle to print inks with high viscosity (typically >20 mPa·s) and inks containing submicron-scale particles (typically >200 nm) with high density.^5^

Laser-induced forward transfer (LIFT), in which laser-induced cavitation processes project an irradiated donor material and deposit it onto a substrate, has been widely investigated as an alternative nozzle-free material deposition technique.^6^ It has the capacity to directly print high viscosity and high-density donor materials, such as metals, organics, and biomaterials.^7–10^ A Gaussian laser beam with a planar wavefront is typically used in conventional LIFT processes, and this can result in the explosion of donor materials due to localized heating by the central intensity maxima of the beam. As such, the LIFT technique also struggles to directly print donor materials with high spatial resolution at long working distances.^11–13^

An optical vortex exhibits a doughnut-shaped spatial intensity profile and has orbital angular momentum (OAM) (characterized by an integer l ) which is associated with its helical wavefront.^14–17^ In recent years, we have demonstrated a next generation LIFT technology utilizing optical vortex beams instead of Gaussian laser beams. In this case, the OAM of the irradiating optical vortex beam twists the donor material to directly launch a spinning microdroplet with a straight trajectory. This enables the direct print of dots with an ultrahigh spatial resolution at an extremely long working distance.^18^ We herein refer to this technique as OV-LIFT. We have successfully demonstrated the direct print of gold metallic and magnetic nanoinks, dye doped organic solutions (rhodamine B in 1,5-pentanediol), and a colloidal suspension of monodisperse polystyrene nanoparticles with high spatial resolution and high positional accuracy.^19–22^ Also, we have discovered that OV-LIFT enables the close-packing (or crystallization) of metallic, magnetic and dielectric nanoparticles, something which cannot be achieved using conventional LIFT and inkjet technologies.^23^

Cyanobacteria cells, one of the first organisms on earth, produce energy by oxygenic photosynthesis, and they are rapidly finding utility as a light-harvesting biomaterial in advanced applications such as biosolar and bionitrogen fixation devices.^24–26^ Printing of cyanobacteria cells in two- and/or three-dimensions will enable new innovations in the development of light-harvesting devices formed of biomaterials. However, the direct print of biomaterials via OV-LIFT has yet to be demonstrated, and its viability is still in question.

In this paper, we report on the first demonstration of direct printing of cyanobacteria cells in a colloidal suspension by utilizing OV-LIFT. The printed dots were well aligned at a high positional accuracy of <8 μm. Interestingly, the cyanobacteria cell viability (∼90%) in the printed dots is significantly higher than that (∼63%) obtained by using conventional LIFT with a Gaussian laser.

## 2 Method and Donor Preparation

Figure 1a shows a schematic diagram of the experimental setup used for the direct print of cyanobacteria cells. Unicellular cyanobacteria cells (cell size: 1.7 μm) in suspension (a mixture of 70 wt % glycerol (C_3_H_8_O_3_) and 30 wt % purified water (H_2_O)) was used as a donor material. The cell concentration and viscosity were estimated to be 8.4 × 10^11^ cells/L and 140 mPa·s, respectively. The suspension exhibited a broad absorption band around 450 nm and strong emission around 614 nm (arising from chlorophyll) under 488 nm excitation as shown in Figure 1b. The suspension was dropped onto on an ultraviolet (UV) cleaned microscopic glass substrate to form the donor film. To avoid the donor dripping associated from gravitation effects, the UV cleaned microscopic glass receiver was then placed on top of the donor film. Note that the high-viscosity donors such as solids and paste like materials can be projected downward by OV-LIFT.

**Figure 1:**
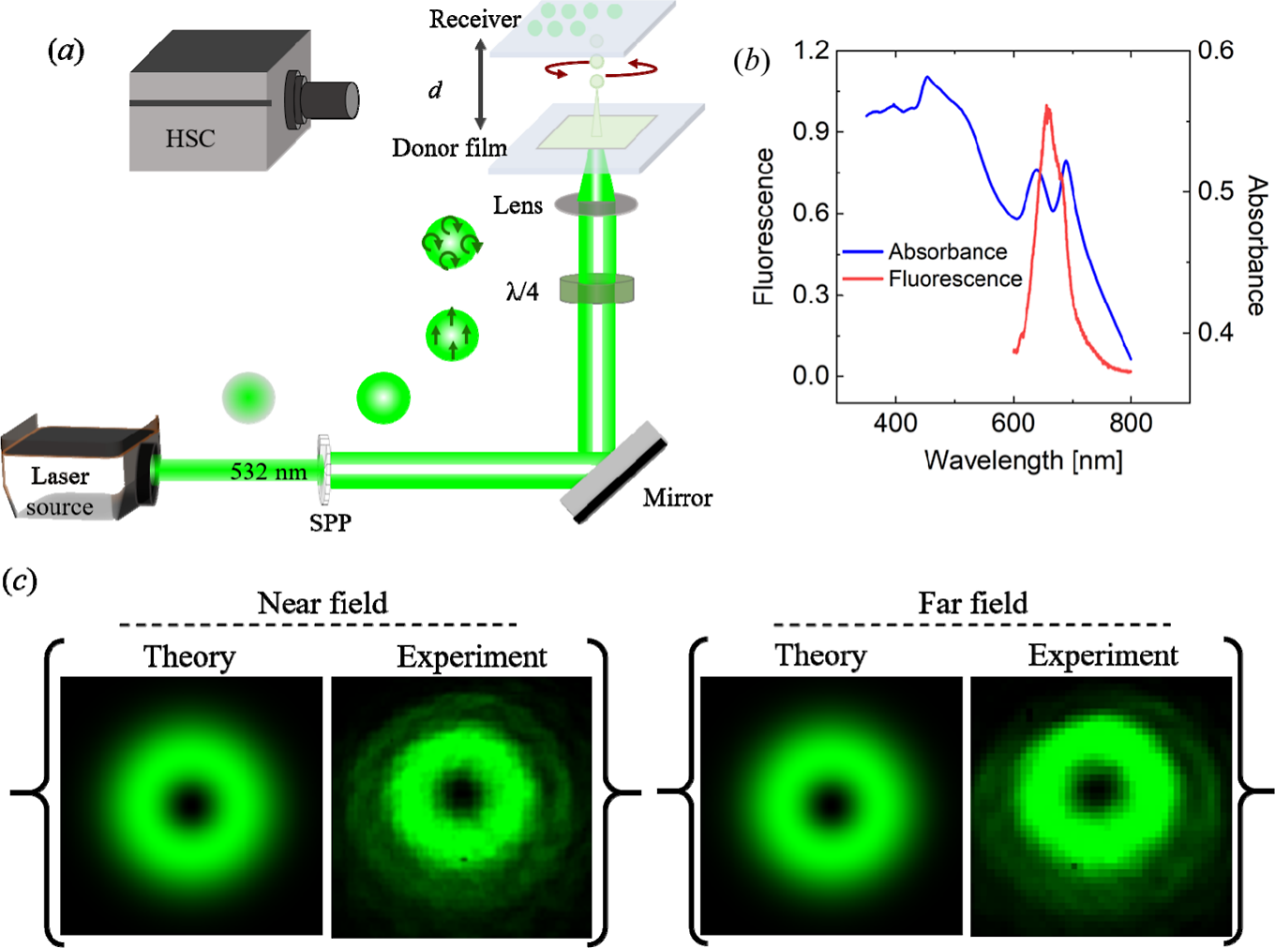
(a) Schematic diagram of the OV-LIFT experimental setup used for cyanobacteria printing. Spiral phase plate (SPP), quarter-wave plate (λ/4), working distance (*d*) and high-speed camera (HSC). (b) Plot of the absorption and emission spectra of cyanobacteria cells in solution. (c) Theoretical and experimental near- and far-fields of optical vortex.

A nanosecond pulsed green laser (wavelength: 532 nm, pulse duration: ∼10 ns, repetition rate: 50 Hz) was used as the laser source. Its output was converted into a circularly polarized first order optical vortex beam by using a spiral phase plate and a quarter-wave plate. It should be mentioned that both the polarization and wavefront helicities of the optical vortex possessed the same sign; namely the total angular momentum (TAM), given by the sum of the spin angular momentum (SAM) associated with circular polarization and the OAM, was 2ℏ. A 2.5 mm annular spot of single optical vortex pulse was focused to be an annular spot (which had a diameter of 39 μm) onto the donor film by using a single plano-convex lens (numerical aperture (NA) ∼0.05, focal length *f* = 25 mm) [Figure 1c]. A single droplet was then ejected from the donor film to form a dot on the receiver. Temporal evolution of the droplet ejection was captured by using an ultrahigh-speed camera with a frame rate of 200 ns per frame (5 Mfps).

Note that both the donor film and receiver were mounted on an *xy*-translation stage controlled by piezo-actuators (positional accuracy: ∼5 μm, minimum displacement: 100 μm), which enabled 2D printing of donor dots.

## 3 Experiment and Discussion

OV-LIFT with circular polarization enabled high-definition direct printing of one-dimensional dot arrays with very low levels of satellite debris. Printed dots took the form of well-aligned circles with a diameter of 70 ± 7 μm and a positional accuracy of <8 μm (Figure 2). Also, note that positional accuracy of printed dots was limited by the positional accuracy of the piezo-actuator used as a translator in our experiments. Higher-definition direct-print will be possible by the refinements of translators. Interestingly, when the optical vortex was linearly polarized (TAM = 1), the positional accuracy became slightly worse (∼10 μm). As mentioned in our previous publications,^21–23^ this highlights that SAM accelerates the spinning motion of donor droplet owing to constructive SAM-OAM coupling effects in optical vortices with a positive SAM and a positive OAM (SAM-to-OAM conversion effects), thus resulting in that SAM reinforces the straight flightpath of the droplets. Note that the SAM-to-OAM conversion effects never occur in isotropic and homogeneous materials, such as the donor film, under a paraxial condition, when the Gaussian beam without OAM is used.^27^ Thus, the circularly polarized optical vortex enables the high-definition print of dots with the highest position accuracy. The thickness of the donor film was measured to be 30 μm.

**Figure 2:**
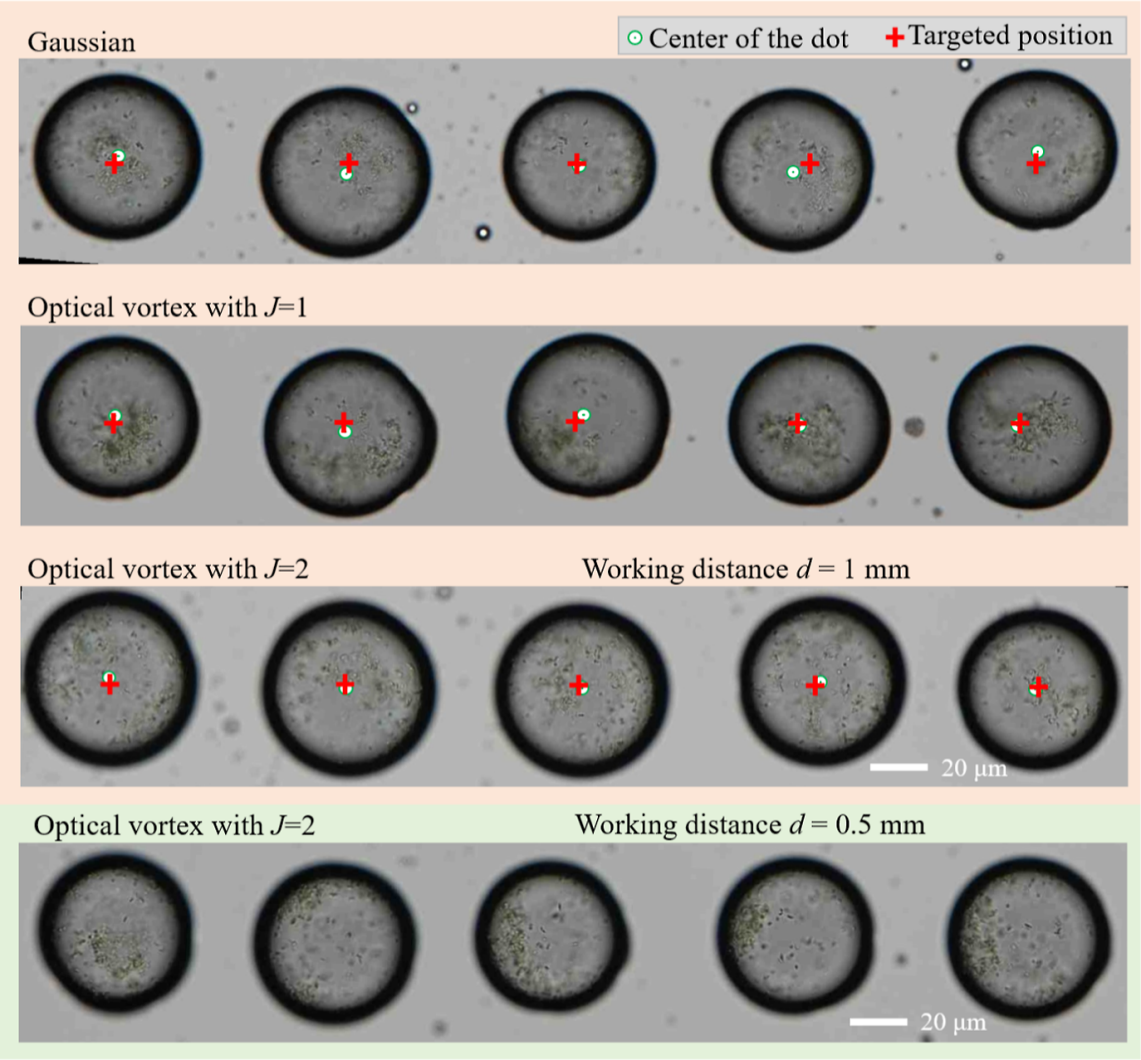
Images showing one-dimensional cyanobacteria cell dot arrays formed using a Gaussian beam (first row), optical vortex beam with linear polarization (second row), optical vortex beam with circular polarization (third row). All three rows are printed at working distance of 1 mm between donor and receiver. The printed dots in the last row correspond to optical vortex beam with circular polarization at working distance of 0.5 mm.

The OV-LIFT allows the high-definition direct print of donor even with a milli-meter scale working distance.^18^ In fact, the direct print of cyanobacteria suspension was demonstrated at working distances (donor–acceptor distance) of 0.5 and 1 mm. The print performances (dot diameter, positional accuracy etc.) were not so sensitive to the working distance. Thus, the working distance was fixed to be 1 mm in this work. The comparison studies for working distances of 0.5 mm and 1 mm are shown in Figure 2.

In contrast, the conventional LIFT (even with a circularly polarized Gaussian beam) process yielded significantly poorer results. The printed dots were nonuniform, and their diameter ranged from 67 to 80 μm. The positional accuracy of the printed dots was typically measured to be ∼16 μm. Also, the Gaussian beam frequently produced many undesired satellites around the printed dots.

The diameter of dots printed using the OV-LIFT technique was almost inversely proportional to the numerical aperture (NA) of the focusing optics (Figure 3); dots with a diameter of ∼19 μm were produced using optics with NA = 0.13. Also, the number of bacteria cells in the printed dots were then measured to be 5. Note that the film thickness was properly controlled within the range of 20–40 μm in this experiment.

**Figure 3:**
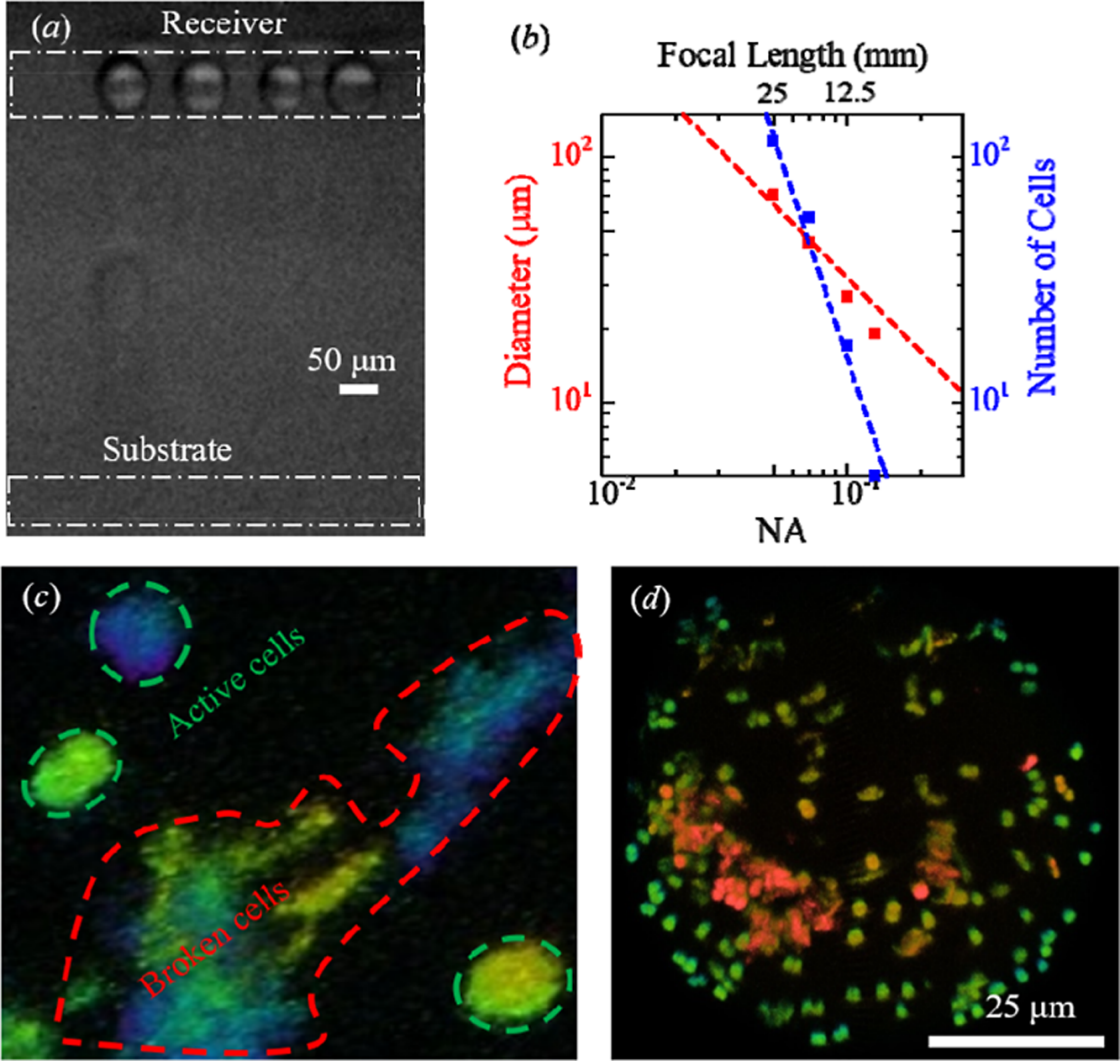
(a) Optical bright image of 1D printed cyanobacteria cell droplets observed using a high-speed camera in OV-LIFT. (b) Plot showing the diameter of printed dots and the number of cyanobacteria cells as a function of numerical aperture of focusing optics. (c) Image showing the morphology of printed dots as observed using laser scanning fluorescence microscopy (dead bacteria cells are highlighted by the red dashed line and active bacteria cells are highlighted by the green dashed line). (d) Confocal laser scanning microscopy image of bacteria cells in a printed dot.

Active bacteria cells exhibit strong red emission under 488 nm laser illumination; this can be used as a visual indicator that cells are alive. Also, dead cells exhibit a broad shape with a fuzzy outline when viewed under a microscope. Thus, the viability of cyanobacteria cells contained within printed dots deposited using LIFT and OV-LIFT were investigated under 488 nm illumination and confocal laser microscopy. Such analysis revealed that dots printed with OV-LIFT exhibited high cell viability (∼90%), while those printed using LIFT had lower cell viability (∼63%). Also, the density of bacteria cells in the dots printed using OV-LIFT was 1.5 times higher than those printed using LIFT. Interestingly, the number of cells contained within dots printed using OV-LIFT was inversely proportional to the NA of the optics. The pulse energy (fluence) of the laser was fixed near the threshold energy for the ejection of a single droplet (minimum energy required for printing) to avoid excessive energy deposition into the cells.

The printed dots including living/active cyanobacteria cells were exposed to white light illumination at room temperature for a period of 40 days. In doing so, the number of cells doubled or tripled (approximately 2.5 times); this demonstrates the high cell viability when using the OV-LIFT technique (Figure 4).

**Figure 4:**
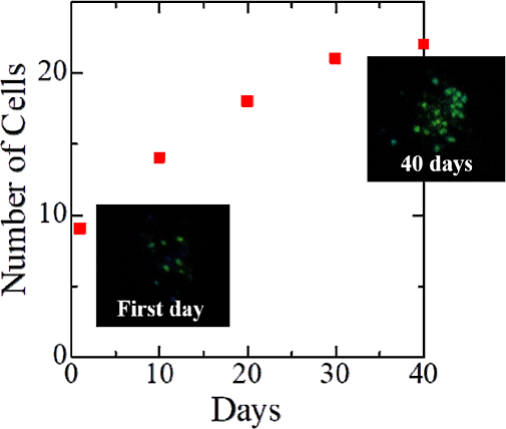
Postprinting growth of cyanobacteria cells under white light illumination. The plots show the number of bacteria within a single printed dot as a function of days. Confocal laser microscope images of a printed dot on the first day and on 40th day under white light illumination are shown in the inset.

Irradiation of the donor film with a laser pulse produces a cavitation bubble in the irradiation area. The temporal evolution of this laser-induced cavitation bubble when using both a Gaussian and optical vortex beam was examined using a high-speed camera (Figure 5). It was observed that the optical vortex induced cavitation bubble started to shrink rapidly at ∼3.2 μs after arrival of the laser pulse. However, in the case of irradiation using a Gaussian beam, the induced cavitation bubble then kept growing. The rapid shrinking of the optical vortex induced cavitation bubble produces an inward (negative) pressure within the donor film and this acts to confine cyanobacteria cells with high density, as mentioned in our previous publications.^18^

**Figure 5:**
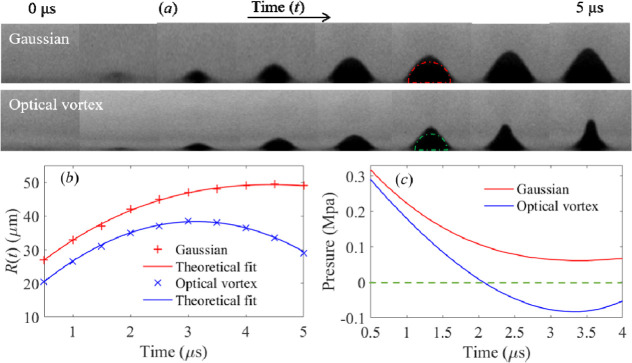
(a) Sequence of images showing the temporal evolution of blister formation on the donor film in a time period of 0–5 μs following illumination by a Gaussian (upper panel) and an optical vortex (lower panel) pulse. The blister outline is fitted with a semicircle function (as highlighted by the broken red and green lines) to estimate the cavitation bubble radius. (b) Plots showing the temporal evolution of the cavitation bubble radius generated by illumination of both Gaussian and optical vortex pulses. (c) Plots show the estimated cavitation pressure outward (positive) and inward (negative) of the blister under illumination by Gaussian and optical vortex beams, as modeled using the Rayleigh–Plesset equation. Zero pressure is indicated by the green dashed line.

To quantitatively understand the temporal evolution of cavitation bubble, Rayleigh–Plesset analysis was conducted.^28^

Assuming that the blister reflects the temporal evolution of the cavitation bubble, the cavitation bubble radius *R*(*t*) was then estimated by fitting a blister with a hemisphere function.

The Rayleigh–Plesset equation is given as follows^29^

1 $$\frac{P_{B}\left(t\right)-P_{\infty }\left(t\right)}{\rho_{L}}=R\left(t\right)\frac{d^{2}R\left(t\right)}{dt^{2}}+\frac{3}{2}{\left(\frac{dR\left(t\right)}{dt}\right)}^{2}+\frac{4v_{L}}{R\left(t\right)}\frac{dR\left(t\right)}{dt}+\frac{2S}{\rho_{L}R\left(t\right)}$$

where *P*_B_(*t*) is the cavitation pressure within the bubble, *P*_∞_(*t*) (∼0.1 MPa) is the pressure at an infinite distance outside the bubble, *R*(*t*) is the cavitation bubble radius, ρ_L_ (=∼1180 kg/m^3^) is the donor density, ν_L_ (=∼1.91 × 10^–5^ m^2^/s) is the dynamic viscosity, and *S* (∼0.0662 N/m) is the surface tension.^30,31^

The optical vortex induced cavitation pressure (reaching a maximum value of ∼0.29 MPa) decreases rapidly. The optical vortex produces a negative (inner compression) cavitation pressure (−0.08 MPa < atmospheric pressure (0.1 MPa)) in the donor blister at ∼3.4 μs after laser irradiation. Rapid shrinkage of the cavitation bubble results in high density confinement of cyanobacteria cells (117). Also, this moderate (inner compression) cavitation pressure, which is less than atmospheric pressure, maintains high cell viability.

In contrast, a Gaussian beam (as used in LIFT) produced high positive (outward expansion) pressure (0.06–0.32 MPa), which resulted in low density confinements of cells (81) in the printed dots and lower cell viability.

The OV-LIFT process was also used to demonstrate direct printing of 2-dimensional (2D) dot arrays (average diameter: 51 ± 6 μm, positional accuracy: ∼7.6 μm) without satellite debris (Figure 6) (NA of focusing optics ∼0.07).

**Figure 6:**
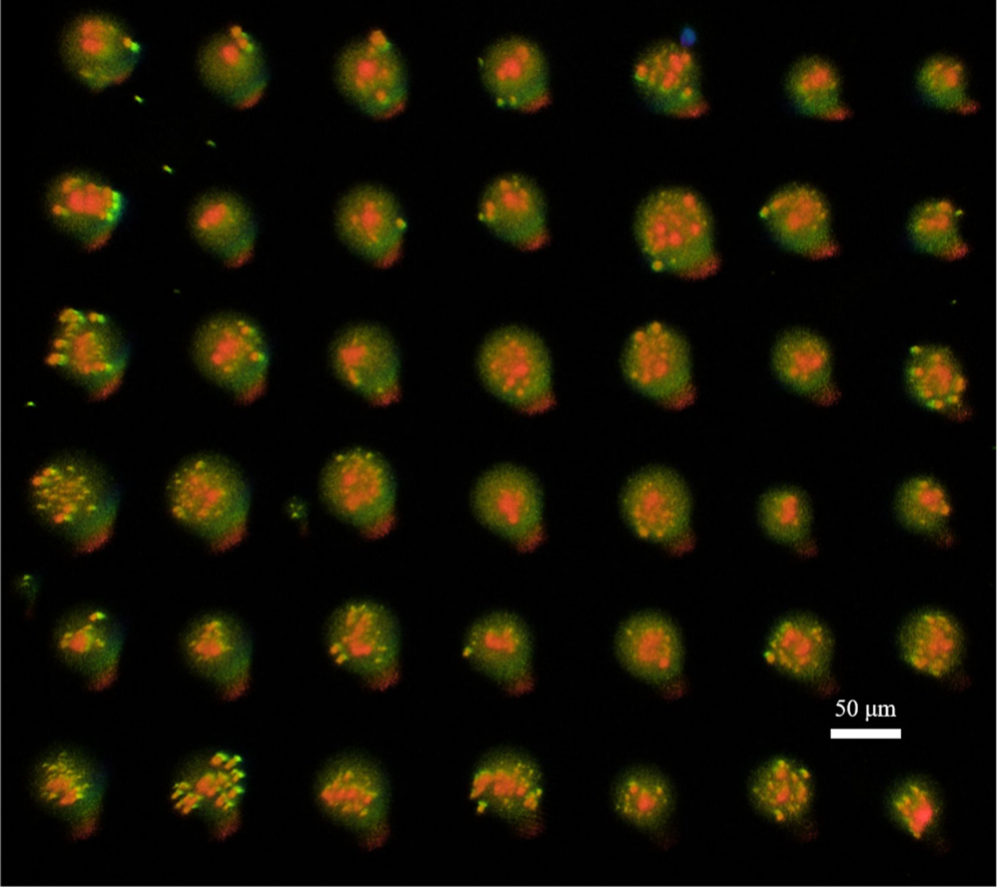
Image of a 2D cyanobacteria suspension dot array printed using OV-LIFT. Numerical aperture of focusing optics is 0.07.

## 4 Conclusion

We have demonstrated the direct print of 2D biomaterial (cyanobacteria cells in a colloidal suspension) dot arrays by using the OV-LIFT technique. The positional accuracy of the printed dots was <8 μm.

Interestingly, the survival rate (∼90%) of cyanobacteria cells with oxygenic photosynthesis in dots printed by OV-LIFT was significantly higher than that (∼63%) obtained by conventional LIFT. The viability of printed cells was confirmed by using a postprinting growth method wherein the number of cells doubled after 40 days. We anticipate that this methodology will enable the fabrication of freeform two- or three-dimensional printed microstructures for biosolar cell devices or cell scaffolds for tissue engineering.
